# Intravoxel incoherent motion (IVIM) in diffusion-weighted imaging (DWI) for Hepatocellular carcinoma: correlation with histologic grade

**DOI:** 10.18632/oncotarget.12689

**Published:** 2016-10-15

**Authors:** Vincenza Granata, Roberta Fusco, Orlando Catalano, Benedetta Guarino, Francesco Granata, Fabiana Tatangelo, Antonio Avallone, Mauro Piccirillo, Raffaele Palaia, Francesco Izzo, Antonella Petrillo

**Affiliations:** ^1^ Department of Radiology, “Istituto Nazionale Tumori IRCCS Fondazione Pascale – IRCCS di Napoli”, Naples, I-80131, Italy; ^2^ Department of Civil and Mechanical Engineering, “Università degli Studi di Cassino e del Lazio Meridionale”, Cassino 03043, Italy; ^3^ Departement of Pathology, “Istituto Nazionale Tumori IRCCS Fondazione Pascale – IRCCS di Napoli”, Naples, I-80131, Italy; ^4^ Department of Gastrointestinal Oncology, “Istituto Nazionale Tumori IRCCS Fondazione Pascale – IRCCS di Napoli”, Naples, I-80131, Italy; ^5^ Department of Hepatobiliary Surgical Oncology, “Istituto Nazionale Tumori IRCCS Fondazione Pascale – IRCCS di Napoli”, Naples, I-80131, Italy

**Keywords:** HCC, magnetic resonance imaging, diffusion weighted imaging, histologic grade

## Abstract

**Purpose:**

To assess the correlation between DWI diffusion parameters obtained using Intravoxel Incoherent Motion Method (IVIM) and histological grade of Hepatocellular carcinoma (HCC).

**Results:**

According to Edmondson-Steiner grade lesions were classified with grade 1 (14), grade 2 (30), grade 3 (18), and grade 4 (0). Apparent Diffusion Coefficient (ADC), perfusion fraction (fp), tissue diffusion coefficient (Dt) median values were statistically different in HCC groups with 1, 2, 3 histological grade (p<0.001). A significant correlation was reported between ADC, fp, Dt and histologic grade respectively of 0.687, 0.737 and 0.674. Receiver operating characteristic (ROC) analysis demonstrated that an ADC of 2.11×10-3 mm2/sec, an fp of 47.33% and an Dt of 0.94×10-3 mm^2^/sec were the optimal cutoff values to differentiate high histological grade (3) versus low histological grade (1-2), with a sensitivity and specificity for ADC of 100% and 100%, for fp of 100% and 89%, for Dt of 100% and 74%, respectively.

**Material and Methods:**

A retrospective approved study was performed including 34 patients with 62 HCCs. IVIM was performed to obtain ADC, fp, pseudo-diffusion coefficient (Dp), Dt coefficients. Kruskal Wallis, Spearman Correlation Coefficient, ROC analysis were performed.

**Conclusions:**

ADC and IVIM-derived fp showed significantly better diagnostic performance in differentiating high-grade from low-grade HCC, and significant correlation was observed between ADC, fp, Dt and histological grade.

## INTRODUCTION

Hepatocellular carcinoma (HCC) is one of the most common human solid tumors worldwide [1, 2]. European Association for the Study of the Liver (EASL), in accordance with the guidelines of the American Association for the Study Liver Diseases (AASLD), recommended that in order to characterize HCC, invasive criteria by imaging techniques such as multidetector Computed Tomography (CT) and Dynamic Contrast enhanced Magnetic Resonance Imaging (DCE-MRI) can be utilized only in cirrhotic patients [3]. Diagnosis should be based on the identification of the typical hallmark of HCC (hypervascular in the arterial phase with washout in the portal venous or delayed phases) [3]. Although the MRI is the technique which best suits to characterize and to follow HCC patients [4, 5], no agreement regarding the study protocol and the impact of Diffusion-weighted imaging (DWI) has been found. DWI is capable of evaluating the mobility of water proton and can provide information on microstructural organization of a tissue, the cell density, the integrity of cell membrane and its vitality [6, 7]. During 1986, Le Bihan, was the first, to evaluate intravoxel incoherent motion (IVIM) as an analytical approach to characterize the link between the tissues signal attenuation and the increase in the b value, and thanks to biexponential model, it was possible to separate the diffusion of water molecules to microcapillary perfusion of tissues [8]. IVIM data can be evaluated either quantitatively or qualitatively; quantitative data may be useful for tissue characterization and functional assessment, while qualitative evaluation may be useful to identify disease [9]. In clinical practice, DWI is used to assess treatment response of liver lesions after neoadjuvant therapy [10] or ablative techniques [11]. Another interesting field is the correlation between the quantitative and semi-quantitative values of DWI with histological grade of the tumor, as was seen in bladder [12], endometrial [13], breast [14], and brain cancers [15]. Recent studies, also in HCC, had evaluated the correlation between DWI and histological grade [16–19] considering that the HCC histologic grade is an important recurrence and survival predictive factor after hepatic resection and transplantation: poorly differentiated HCC are associated to worse survival in comparison of well and moderately differentiated HCC [20]. The possibility that imaging study can correlate to histologic grade to select the therapeutic strategy would be of great value in helping to direct the proper management of HCC. Therefore, our purpose was to test if DWI and IVIM parameters could correlate with histological grade of HCC and became a prognostic imaging factors.

## RESULTS

**Figure 1:**
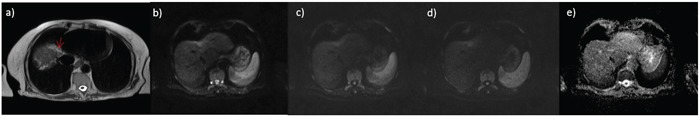
small HCC on IV hepatic segment G1; in **a.** HASTE T2-wejgthed image, in **b.** DWI image at b value 50 s/mm^2^, in **c.** DWI image at b value 400 s/mm^2^, in **d.** DWI image at b value 800 s/mm2 and in **e.** ADC map.

**Figure 2:**
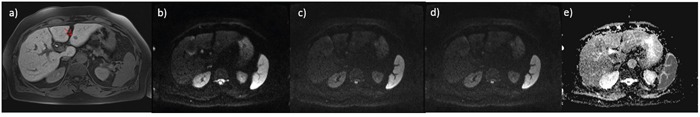
small HCC on III hepatic segment G2; in **a.** VIBE T1 weighted image, in **b.** DWI image at b value 50 s/mm^2^, in **c.** DWI image at b value 400 s/mm^2^, in **d.** DWI image at b value 800 s/mm^2^ and in **e.** ADC map.

**Figure 3:**
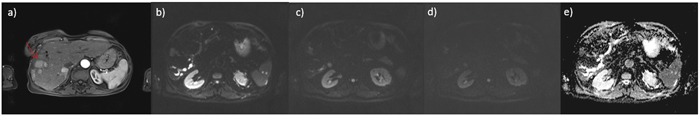
HCC on V hepatic segment G3; in **a.** VIBE T1 weighted image, in **b.** DWI image at b value 50 s/mm^2^, in **c.** DWI image at b value 400 s/mm^2^, in **d.** DWI image at b value 800 s/mm^2^ and in **e.** ADC map.

A total of 62 HCCs were evaluated (median 1.82 lesions; diameter between 12 and 20 mm). All tumors were histologically classified according to the major Edmondson-Steiner grade on final pathologic reports as follows: 14 with grade 1, 30 with grade 2, 18 with grade 3, and no one with grade 4. Figure 1, 2 and 3 report example of HCC of grade 1, 2 and 3 respectively.

Table 1 reports mean value ± standard deviation for ADC ad IVIM parameters for different histological grade. Mean values of ADC, fp, Dt were statistically different in HCC groups with 1, 2, 3 histological grade (p value <0.001 at test of Kruskal Wallis).

**Table 1:** ADC and IVIM derived diffusion and perfusion parameters: mean and standard deviation values according to different histological grade of HCC

Grading		ADC [mm^2^/s 10^−3^]	fp [%]	Dp [mm^2^/s 10^−3^]	Dt [mm^2^/s 10^−3^]
**1**	Mean	**0,94**	**14,48**	**54,69**	**0,86**
	Standard deviation	0,12	7,38	83,62	0,09
**2**	Mean	**1,03**	**31,49**	**44,49**	**0,81**
	Standard deviation	0,31	17,60	70,15	0,49
**3**	Mean	**2,44**	**54,08**	**13,70**	**1,51**
	Standard deviation	0,04	2,96	7,39	0,11
**Total**	Mean	1,42	34,21	36,78	1,03
	Standard deviation	0,70	19,25	61,45	0,46
P value*		**<0.001**	**<0.001**	0.385	**<0.001**

* Kruskal-Wallis Test

Correlation coefficient obtained according to Spearman's rank was performed for each couple of parameters and was reported in Table 2. A significant correlation was reported between ADC, fp, Dt and histologic grade of HCC respectively of 0.687, 0.737 and 0.674 with a significant statistically difference (p value < 0.05).

**Table 2:** ADC and IVIM derived diffusion and perfusion parameters: Spearman Correlation Coefficient for each couple of parameters

			GRADING	ADC	fp	Dp	Dt
***Spearman Correlation Coefficient***	**GRADING**	Correlation Coefficient	1,000	,687*	,737*	−,326	,674*
		P value		,000	,000	,090	,000
	**ADC**	Correlation Coefficient	,687*	1,000	,483*	−,128	,835*
		P value	,000		,006	,518	,000
	**fp**	Correlation Coefficient	,737*	,483*	1,000	−,615*	,245
		P value	,000	,006		,001	,183
	**Dp**	Correlation Coefficient	−,326	−,128	−,615*	1,000	,290
		P value	,090	,518	,001		,135
	**Dt**	Correlation Coefficient	,674*	,835*	,245	,290	1,000
		P value	,000	,000	,183	,135	

* A p value < 0.05 was considered statistically significant.

ROC analyses demonstrated that an ADC of 2.11×10^−3^ mm^2^/sec, an fp of 47.33% and an Dt of 0.94 x10^−3^ mm^2^/sec were the most accurate cutoff levels (calculated with Youden index) to differentiate high histological grade (3) versus low histological grade (1 and 2), with a sensitivity and specificity for ADC of 100% and 100%, for fp of 100% and 89%, for Dt of 100% and 74%, respectively.

In addition, ADC was shown to have the best diagnostic performance in comparison of fp and Dt for differentiating high from low grade of HCC (grade 3 versus and 2), with a corresponding area under the ROC curve of 1.0 (p value <0.001), 0.90 (p value = 0.05) and 0.95 (p value = 0.036) (Table 3).

**Table 3:** ADC and IVIM derived diffusion and perfusion parameters: Area under ROC curve with optimal cutoff values, sensitivity and specificity

	Area	Cutoff value	pvalue	Sensitivity [%]	Specificity [%]
**ADC [mm^2^/sec]**	1,000	2.11×10^−3^	,000	100	100
**fp [%]**	,895	47,33	,000	100	89
**Dp [mm^2^/sec]**	,327	18.3 x10^−3^	,038	56	43
**Dt [mm^2^/sec]**	,947	0.94 x10^−3^	,000	100	74

## DISCUSSION

The accurate distinction of well-differentiated HCCs from less well-differentiated HCCs is considered an important issue in planning the therapeutic strategy [23]. Considering that histological confirmation of small suspicious hepatic nodules before treatment is often not possible owing to their location in the liver, the role of pre-operative imaging technique for the discrimination of well, moderate and poorly differentiated HCCs is important [24]. Our results suggested that DWI could be used to predict the histological grade of HCC, in fact there was a good correlation between ADC and grading, between fp and grading, and between Dt and grading. Nakanishi et al, in previous study, demonstrated not only the usefulness of DWI for histological tumor grading, but also the possibility to use ADC as an early predictive factor in pre-surgical phase of HCC recurrence in the six months following surgery [25]. DWI is a valuable diagnostic tool, that using water mobility assessment reflects indirectly tissue biological characteristics [12–19, 25]. Water mobility is restricted in malignant tissue due to increase of cellular density and decrease of interstitial space; this translates to signal hyperintensity in diffusion weighted images and signal hypointensity in an ADC map [7]. In a recent meta-analysis was reported the DWI performance in the prediction of HCC histological grade: Chen et al showed that in the discrimination between well differentiated HCC by higher grades, DWI had a sensitivity of 54%, a specificity of90%, and an area under ROC curve 0.9311. While, for differentiating poorly differentiated HCC from lower grades, the sensitivity was 84%, specificity was 48%, and a moderately high diagnostic performance (AUC = 0.8513) [18]. According to Chen, in our study, ROC analyses demonstrated that an ADC of 2.11×10^−3^ mm^2^/sec, an fp of 47.33% and an Dt of 0.94×10^−3^ mm^2^/sec were the most accurate cutoff levels to differentiate high histological grade (3) versus low histological grade (1 and 2), with a sensitivity and specificity for ADC of 100% and 100%, for fp of 100% and 89%, for Dt of 100% and 74%, respectively. Nasu et al, in a series of 125 resected HCCs (sizes range: 0.8–15 cm), found no correlation between histological grade and ADC (using b factors of 0 and 500 smm2), although the DWI and Signal Intensity (SI) of the HCCs increased in higher grade [26]. Instead, Muhi et al showed significant differences both in SI and ADC values (using b factors of 500 and 1000 smm2) between various grades of 98 HCC (size range, 0.8–5.3 cm), although there was still considerable overlapping [27]. Nishie et al demonstrated a correlation of ADC value with the HCCs histological grade, but the difference was significant only between the extreme groups (between well-differentiated HCCs and poorly differentiated HCCs) [28]. Conversely, to Nishie, in our series, there was a linear proportional tendency for ADC, fp and Dt values according to the transition from well-differentiated to poorly differentiated. Current technique improvement has supported the IVIM usein the prediction of the HCC histological grade. IVIM approach provides both estimate of water pure mobility and a measure of microscopic blood diffusion in capillaries network. [8–9]. Pure diffusion coefficient (Dt) derived by IVIM model according to Woo et al had significantly better diagnostic accuracy than apparent diffusion coefficient to differentiate low and high HCC histological grade [17]. Conversely in our study, the best diagnostic performance was obtained by ADC in comparison of fp and Dt for differentiating high from low grade HCC (grade 3 versus 1 and 2), with a corresponding area under the ROC curve of 1.0 (p value <0.001), 0.90 (p value = 0.05) and 0.95 (p value = 0.036).

This study has a limitation. To obtain accurate informations about tissue perfusion proprieties by IVIM model applied on DWI data in need to acquire imaging using lower b values (< 100–200 s/mm^2^) and an adequate total number of b values. Conversely, there is no consensus on number and amplitude of the b values that should be used in clinical setting. The magnitude of b values differs between literature studies and the number ranges from 4 to more than 10. On the other hand, different b-values groupings may cause bias and variability estimation of IVIM parameters, especially in IVIM-derived fp [29]. Koh et al [30] suggested six to eight b values in total, with four or more within the perfusion-sensitive range (b < 100 s/mm^2^) to better quantify pseudo-diffusion motion. Due to the restriction of the used MR equipment which does not allow to use b values with an interval of less than 50 ms, we have utilized only 3 b values between 0 and 100 (0, 50 and 100 s/mm^2^). To obviate this constraint we used a special algorithm to calculate IVIM parameters that Fusco et al [22] demonstrated to perform better than conventional Levenberg Marquardt (LM) algorithm for IVIM parameters estimation. Fusco et al in [22] examined the performance of LM and VARPRO for IVIM estimation on a simulation basis and concluded that VARPRO technique had smaller bias and better fitting with respect to LM; this latter was demonstrated on simulation data in term of residual sum of square (RSS) both for single search start point and for multiple search start point approach.

HCC histologic grade is a important factor to predict recurrence and survival after hepatic resection and transplantation. The possibility that imaging study can correlate to histologic grade to selecting the therapeutic strategy would be of value in helping to direct the proper management in patients with HCC. In this context, MR study with the DWI sequences is the method of choice because the ADC and IVIM-derived fp values is related to the histological grade of HCC, presented a better diagnostic performance to differentiate low and high HCC histological grade.

## MATERIALS AND METHODS

### Patient population

A retrospective study, approved by National Cancer Institute Pascale Foundation of Naples, was performed through a computerized search of medical records on 74 patients underwent liver MR imaging and followed by biopsy for HCC from August 2014 to February 2016. After reviewing the medical records, 18 patients were excluded because the final pathology report was confirmed not to be HCC; 14 patients because the tumors were smaller than 1 cm and 8 were excluded due to patient movement or image noise.

The final study population included 34 patients (8 women and 26 men; mean age 72 years; range: 56-83 years) with 62 HCCs. The mean interval standard deviation between pathologic examination and MR imaging was 15 days (range 4–28 days). All patients had chronic liver disease which was related to hepatitis C virus infection in 14 patients, hepatitis B virus in 18 cases, and alcohol abuse in 2; all were stage A according to the Barcelona-Clinic Liver Cancer (BCLC) classification; alpha-fetoprotein levels were >4 ng/ml (12-320 ng/ml, mean 80 ng/ml) in all patients.

### MR imaging protocol

MR imaging was performed by using a 1.5 T MR (Magnetom Symphony, with Total Imaging Matrix Package, Siemens, Erlangen, Germany) with 8-element body and phased array coils. The MRI examination consisted of basal images taken before IV administration of contrast medium and then functional dynamic sequences obtained after IV injection of liver-specific contrast medium, acquiring the last series of images with a delay of 20 minutes during the hepatobiliary excretion of the contrast medium. The baseline sequences obtained before IV contrast medium were coronal Trufisp T2-weighted free breathing; axial Half-Fourier Acquisition Single-Shot Turbo Spin-Echo (HASTE) T2-weighted, with controlled respiration, without and with fat-suppressed (FS) gradient-echo pulse; coronal HASTE T2-weighted, without FS; axial flash in-out phase T1-weighted, with controlled respiration; Volumetric Interpolated Breath-hold Examination (VIBE) T1-weighted SPAIR with controlled respiration**;** diffusion weighted imaging (DWI) with planar echo-pulse sequence (EPI) at several b value *b* value 0, 50, 100, 200, 400, 600, and 800 s/mm^2^. As contrast medium the liver-specific gadolinium ethoxybenzyl dimeglumine (Primovist, Bayer Schering Pharma, Germany) was employed All patients received 0.1 ml/kg of Primovist by means of a power injector (Spectris Solaris® EP MR, MEDRAD Inc., Indianola, IA, USA), at an infusion rate of 1 ml/s. After completion of IV contrast medium administration, VIBE T1-weighted FS (SPAIR) sequences were acquired in five different phases: hepatic arterial (35 s delay), portal venous (70 s), equilibrium (90 s), delayed (120 s), hepatobiliary excretion (20 minutes). Details of sequence parameters were reported in Table 4 [10, 21].

**Table 4:** Pulse Sequence Parameters on MR studies

Sequence	Orientation	TR/TE/FA (ms/ms/deg.)	AT (min.)	Acquisition Matrix	Slice thickness/Gap (mm)	Fat Suppression
TRUFISP T2-W	Coronal	4.30/2.15/80	0.46	512×512	4/0	without
HASTE T2-W	Axial	1500/90/170	0.36	320×320	5/0	Without and with (SPAIR)
HASTE T2w	Coronal	1500/92/170	0.38	320×320	5/0	without
In-Out phase T1-W	Axial	160/2.35/70	0.33	256×192	5/0	without
DWI	Axial	7500/91/90	7	192×192	3/0	without
VIBE T1-W	Axial	4.80/1.76/12	0.18	320×260	3/0	with (SPAIR)

**Note**.–W= Weighted, TR = Repetition time, TE = Echo time, FA = Flip angle, AT = Acquisition time, SPAIR = Spectral Adiabatic Inversion Recovery, HASTE = Half-Fourier acquisition single-shot turbo spin-echo, DWI = Diffusion-weighted imaging VIBE = Volumetric interpolated breath hold examination

### Images analysis

In this study the diffusion parameters estimation was performed using the intravoxel incoherent motion method [8, 9].

Bi-exponential model to estimate the IVIM-related parameters of pseudo-diffusivity (D_p_), perfusion fraction (f_p_), and tissue diffusivity (D_t_) was described by the following equation

$$\frac{S_{0}}{S_{b}}={\text{f}}_{\text{p}}\text{exp }\left(-b\;D_{p}\right)+\left(1-f_{p}\right)\text{exp}\left(-b\;D_{t}\right)$$

We used a VARiable PROjection approach to estimate the three parameters because the bi-exponential model may often be ill-conditioned because of a limited number of samples, small perfusion fraction and/or similar compartmental diffusivities. In a previous study we have demonstrated that the VARiable PROjection algorithm is superior to the conventional Levenberg–Marquardt algorithm for non linear curve fitting in intravoxel incoherent motion method for DW-MRI data analysis [22]. A brief explanation of VARPRO approach is described in the following.

Rearranging the equation (1) the $S(b)/S_{0}-e^{-bD_{t}}$ is the product of f and a nonlinear function of Dt and Dp:

$$\text{f}({\text{D}}_{\text{p}};{\text{D}}_{\text{t}};\text{b})=S(b)/S_{0}-e^{-bD_{t}}=f(e^{-\text{bDp}}-e^{-bD_{t}})$$

Letting $\text{f}({\text{D}}_{\text{p}};{\text{D}}_{\text{t}}\text{;b})$ the cost functional becomes:

$$S(b)/S_{0}.-e^{-bD_{t}}=||\text{y}-\text{f}({\text{D}}_{\text{p}};{\text{D}}_{\text{t}};\text{b})\text{f}|{|}_{2}$$

Therefore, a separable nonlinear least square model known as VARiable PROjection (VARPRO) can be used to calculate the diffusion parameters. If we knew, the estimate of the nonlinear parameters Dp and Dt the estimate of the linear parameter f could be obtained by:

$$f=e^{-bD_{t}}+\text{y}+\text{f}{\left({\text{D}}_{\text{p}};{\text{D}}_{\text{t}};\text{b}\right)}^{+}$$

where $\text{f}{\left({\text{D}}_{\text{p}};{\text{D}}_{\text{t}}\text{;b}\right)}^{+}$ is the Moore-Penrose generalized inverse of $\text{f}({\text{D}}_{\text{p}};{\text{D}}_{\text{t}}\text{;b})$. Therefore, a new cost functional can be constructed:

$$S(b)/S_{0}.-e^{-bD_{t}}=||\text{y-f}({\text{D}}_{\text{p}};{\text{D}}_{\text{t}};\text{b})\text{f}{\left({\text{D}}_{\text{p}};{\text{D}}_{\text{t}};\text{b}\right)}^{+}\text{y}|{|}_{2}$$

This analysis was ROI-based using median value of single voxel signals for each b value. ROIs for the tumor were manually drawn to include such hyperintense voxels on image at b value 800 s/mm2. No motion correction algorithm was used but ROIs were drawn taking care to exclude areas in which movement artifacts or blurring caused voxel misalignments.

The data analysis was performed using an in-house software written in Matlab (The MathWorks, Inc., Natick, MA, USA).

### Statistical analysis

Data were expressed in terms of median value ± standard deviation. Kruskal Wallis non-parametric test was performed to emphasize significant statistically difference between median value of ADC, Dp, fp and Dt for different grading subgroups.

The Spearman rank correlation test was used to compare the mean ADC and IVIM parameter values according to Edmondson-Steiner grade. The correlation coefficient rho (r) was obtained to compare the degree of correlation as follows: little or no relationship if 0 ≤ r < 0.25, fair if 0.25 ≤ r < 0.5, moderate to good if 0.5 ≤ r < 0.75, and very good to excellent if 0.75 ≤ r.

Receiver operating characteristic (ROC) curves were used to determine whether ADC and IVIM-derived parameters could be used to discriminate between low grade (1-2) and high grade (3) HCC. Then, sensitivity and specificity for predicting high histologic grade were calculated according to the cutoff value that demonstrated the greatest Youden index on estimated curves. The area under the ROC curve was obtained to compare their diagnostic capacities.

A p value < 0.05 was considered statistically significant.

All analyses were performed using Statistics Toolbox of Matlab R2007a (The Math-Works Inc., Natick, MA).
